# Supplementary material for: Expectation violations enhance neuronal encoding of sensory information in mouse primary visual cortex
Source: Nat Commun. 2023 Mar 2;14:1196. doi: 10.1038/s41467-023-36608-8 (PMC9981605; doi:10.1038/s41467-023-36608-8)
Supplement: Supplementary file 1 — Supplementary Information [file 41467_2023_36608_MOESM1_ESM.pdf]

Supplementary information

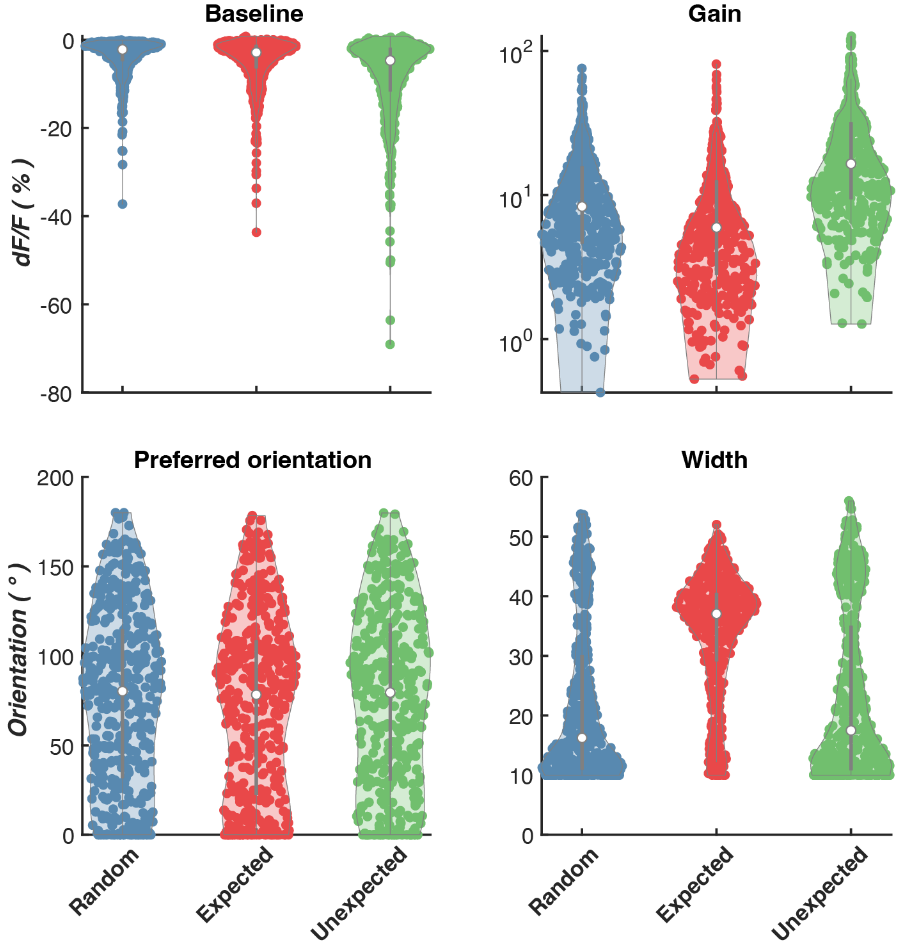

Supplementary Figure 1. *The results of the fitted parameters for the tuning curves which quantify orientation selectivity in the three conditions. See equation 1 for the equation which is fit using non-linear least squared regression. The tuning curves were fit to the 462 orientation selective neurons. Each dot is an individual neuron (n = 462) and the middle white point shows the median. Error bars indicate ±95% confidence interval.*

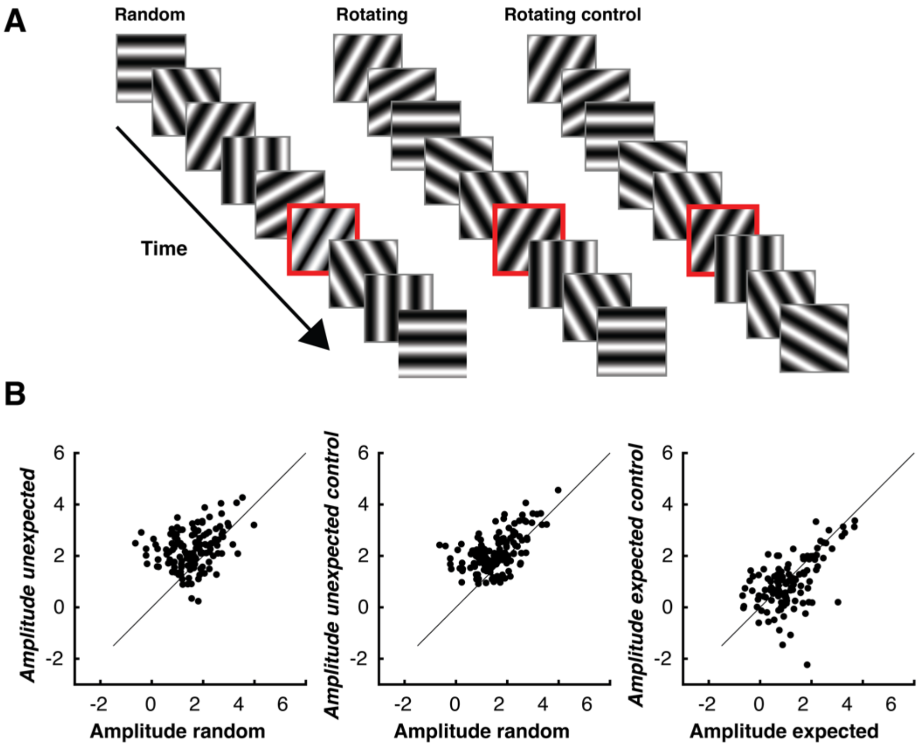

Supplementary Figure 2. *Control condition to determine whether the rotation sequence caused the increased gain in the Unexpected trials.* ***(A).*** *Examples of stimulus sequences for the control experiments.* ***(B).*** *Fitted gain values for each neuron for the three conditions. This subset of neurons showed the same effect in the original two conditions, with an increase in gain in the Unexpected compared to Random condition (t(129 = 7.74, p < 0.001, uncorrected). This effect was maintained when comparing the Random to the Unexpected control condition (t(129) = 7.81 p < 0.001, uncorrected). There was no significant difference between Unexpected and Unexpected control conditions (t(129 = 1.81, p = 0.07, uncorrected).*

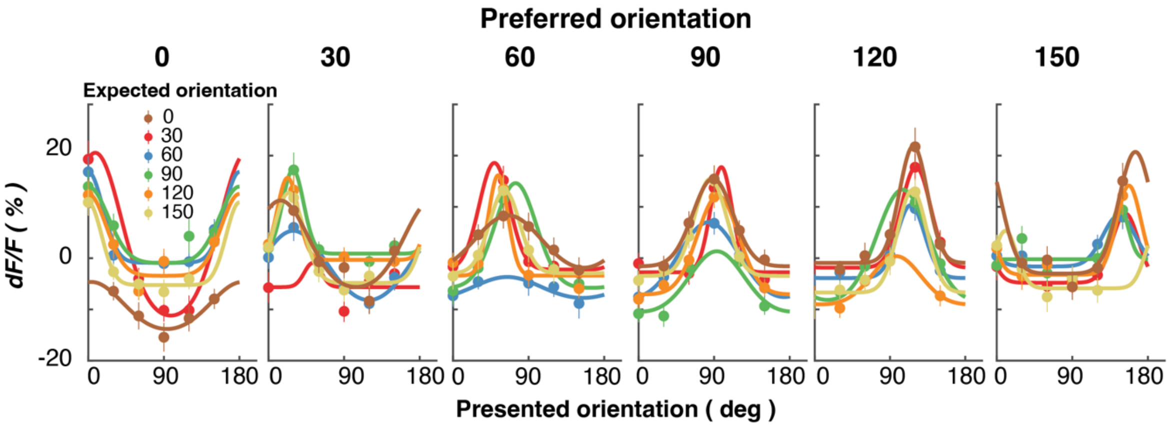

Supplementary Figure 3. *Expectations affect neurons (N = 462) differently depending on their preferred orientation. Each panel shows neurons tuned to different orientations, as defined by their stimulus-evoked responses in the Random condition. The different colour-coded curves show different expected orientations. Neurons show the largest decrease in response when their preferred orientation is similar to the expected orientation. Across all panels error bars indicate ±1 standard error of mean.*

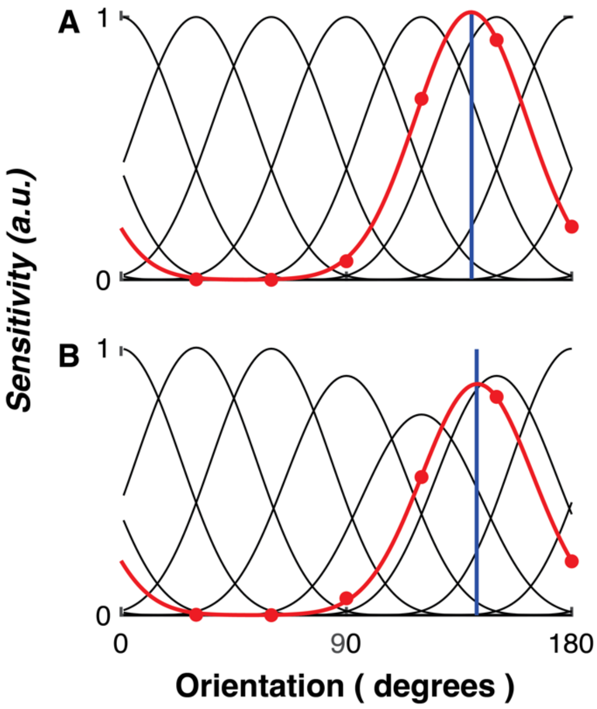

Supplementary Figure 4. *A simple schematic example of the model.* ***(A)*** *The model in an unadapted state, showing its response to a 110° stimulus (blueline). The green line shows the model's response to the stimulus in each channel.* ***(B)*** *Applying adaptation gain at 110° reduces the model's sensitivity to nearby, but not distant, stimuli. The model's response (red line) is reduced in magnitude relative to panel A when the same test stimuli are applied.*

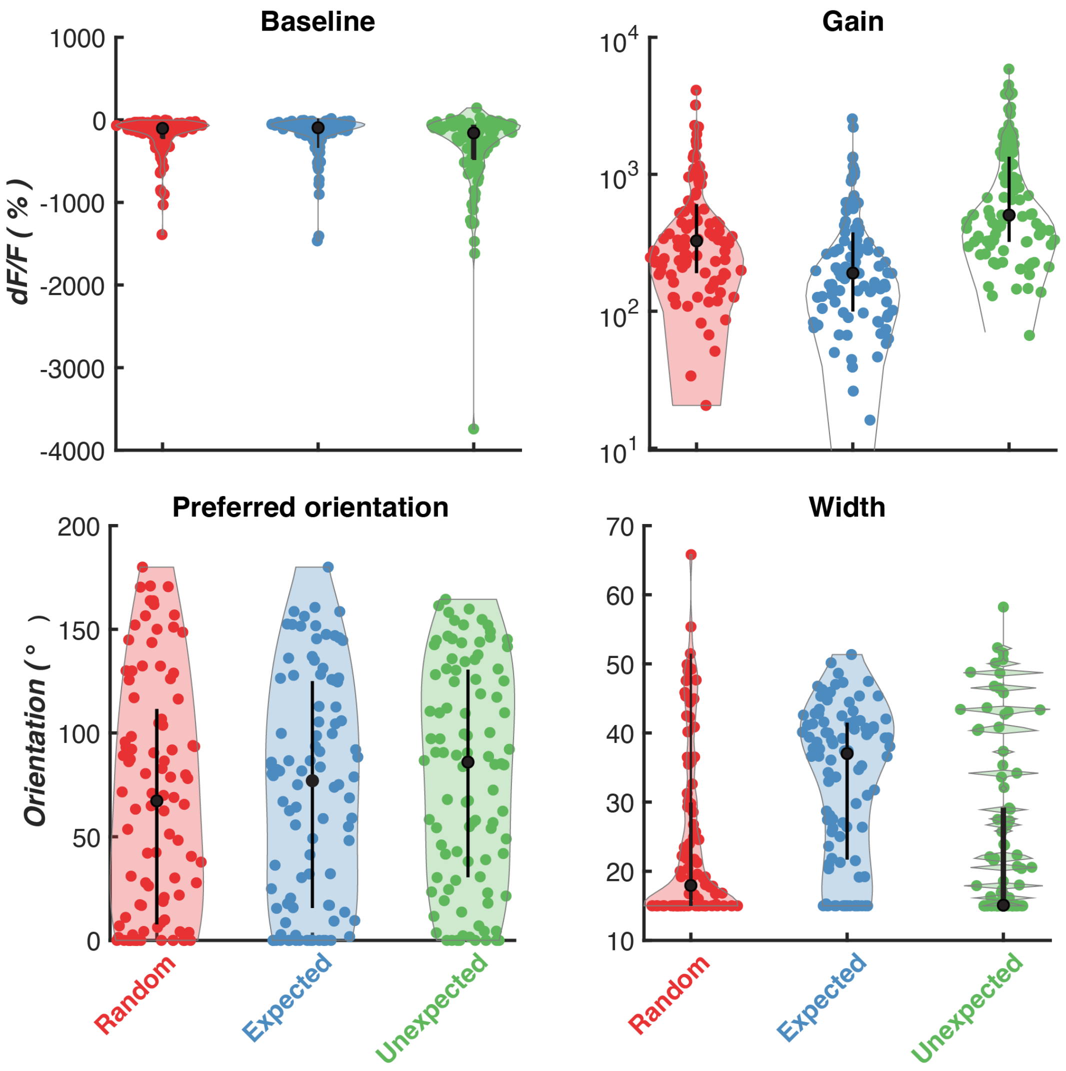

Supplementary Figure 5. *The results of the fitted parameters for the tuning curves which quantify orientation selectivity in the three conditions in the anesthetized experiments. The tuning curves were fit to the 96 orientation-selective neurons in this experiment. This is the same format as Supplementary Figure 1. Error bars indicate the upper and lower quartile range.*
