# Supplementary material for: Expectation violations enhance neuronal encoding of sensory information in mouse primary visual cortex
Source: Nat Commun. 2023 Mar 2;14:1196. doi: 10.1038/s41467-023-36608-8 (PMC9981605; doi:10.1038/s41467-023-36608-8)
Supplement: Supplementary file 3 — Description of Additional Supplementary Files [file 41467_2023_36608_MOESM3_ESM.pdf]

Description of Additional Supplementary Files

File Name: Supplementary Movie 1
Description: Example sequence of gratings in the Rotating condition. Stimuli rotate in one direction for 5 to 9 presentations before jumping to a random orientation and rotating in the opposite direction.
