# Supplementary material for: Expectation violations enhance neuronal encoding of sensory information in mouse primary visual cortex
Source: Nat Commun. 2023 Mar 2;14:1196. doi: 10.1038/s41467-023-36608-8 (PMC9981605; doi:10.1038/s41467-023-36608-8)
Supplement: Supplementary file 5 — Reporting Summary [file 41467_2023_36608_MOESM5_ESM.pdf]

nature portfolio

Corresponding author(s): Matthew Tang

Last updated by author(s): Dec 12, 2022

Reporting Summary

Nature Portfolio wishes to improve the reproducibility of the work that we publish. This form provides structure for consistency and transparency in reporting. For further information on Nature Portfolio policies, see our Editorial Policies and the Editorial Policy Checklist.

Statistics

For all statistical analyses, confirm that the following items are present in the figure legend, table legend, main text, or Methods section.

n/a	Confirmed	
☐	☒	The exact sample size (*n*) for each experimental group/condition, given as a discrete number and unit of measurement
☐	☒	A statement on whether measurements were taken from distinct samples or whether the same sample was measured repeatedly
☐	☒	The statistical test(s) used AND whether they are one- or two-sided<br>*Only common tests should be described solely by name; describe more complex techniques in the Methods section.*
☐	☒	A description of all covariates tested
☐	☒	A description of any assumptions or corrections, such as tests of normality and adjustment for multiple comparisons
☐	☒	A full description of the statistical parameters including central tendency (e.g. means) or other basic estimates (e.g. regression coefficient) AND variation (e.g. standard deviation) or associated estimates of uncertainty (e.g. confidence intervals)
☐	☒	For null hypothesis testing, the test statistic (e.g. *F*, *t*, *r*) with confidence intervals, effect sizes, degrees of freedom and *P* value noted<br>*Give P values as exact values whenever suitable.*
☒	☐	For Bayesian analysis, information on the choice of priors and Markov chain Monte Carlo settings
☒	☐	For hierarchical and complex designs, identification of the appropriate level for tests and full reporting of outcomes
☒	☐	Estimates of effect sizes (e.g. Cohen's *d*, Pearson's *r*), indicating how they were calculated

Our web collection on statistics for biologists contains articles on many of the points above.

Software and code

Policy information about availability of computer code

Data collection	PsychToolbox for MATLAB (R2018) was used for stimulus presentation. The two-photon data were collected using Thorlabs software (ThorImageLS V.3).
Data analysis	We used suite2p (https://github.com/MouseLand/suite2p) to process the two-photon data. All analysis was done using custom MATLAB code. This is available at GitHub (https://github.com/MatthewFTang/PredictionOrientationSelectivityMouseV1)

For manuscripts utilizing custom algorithms or software that are central to the research but not yet described in published literature, software must be made available to editors and reviewers. We strongly encourage code deposition in a community repository (e.g. GitHub). See the Nature Portfolio guidelines for submitting code & software for further information.

Data

Policy information about availability of data

All manuscripts must include a data availability statement. This statement should provide the following information, where applicable:

- Accession codes, unique identifiers, or web links for publicly available datasets
- A description of any restrictions on data availability
- For clinical datasets or third party data, please ensure that the statement adheres to our policy

The data are available at: https://osf.io/t2vb3.

Human research participants

Policy information about studies involving human research participants and Sex and Gender in Research.

Reporting on sex and gender	No human participants were used in this study.
Population characteristics	*Describe the covariate-relevant population characteristics of the human research participants (e.g. age, genotypic information, past and current diagnosis and treatment categories). If you filled out the behavioural & social sciences study design questions and have nothing to add here, write "See above."*
Recruitment	*Describe how participants were recruited. Outline any potential self-selection bias or other biases that may be present and how these are likely to impact results.*
Ethics oversight	*Identify the organization(s) that approved the study protocol.*

Note that full information on the approval of the study protocol must also be provided in the manuscript.

Field-specific reporting

Please select the one below that is the best fit for your research. If you are not sure, read the appropriate sections before making your selection.

☒ Life sciences ☐ Behavioural & social sciences ☐ Ecological, evolutionary & environmental sciences

For a reference copy of the document with all sections, see nature.com/documents/nr-reporting-summary-flat.pdf

Life sciences study design

All studies must disclose on these points even when the disclosure is negative.

Sample size	There were over 1900 neurons included in the study taken from 6 animals in over more than 30 individual recording sessions. Power analysis was not conducted to set the sample size, because no previous work has been conducted using an analogous method so effect size could not be estimated. Instead, the sample size was based on previous work from the lab and the literature which suggests 1900 neurons would provide sufficient power to find small to moderate effect sizes on neuronal tuning using two-photon imaging.
Data exclusions	No data were excluded from the sample
Replication	The number of neurons was very high for the purposes (the main analyses used over 1900 neurons) meaning the statistical power was sufficient. For statistics, we found the effects were reliably found over a large time window, shown using non-permutation cluster analysis. Furthermore, we used a number of independent metrics to show the same of results using all methods. We also replicated these findings in anesthetized animals showing the effects cannot be due to top-down attention states.
Randomization	The study used a completely within-neuron design with the effect of different sensory stimulus conditions on neuronal activity being measured. The conditions were pseudo-randomly presented during the experiment and controlled by a computer.
Blinding	The study used a complete within-subject design with the same neurons examined across conditions. The experimenters were aware of the conditions used in the study but could not predict which condition was presented at any one trial during the experiment.

Reporting for specific materials, systems and methods

We require information from authors about some types of materials, experimental systems and methods used in many studies. Here, indicate whether each material, system or method listed is relevant to your study. If you are not sure if a list item applies to your research, read the appropriate section before selecting a response.

Materials & experimental systems

n/a	Involved in the study	
☒	☐	Antibodies
☒	☐	Eukaryotic cell lines
☒	☐	Palaeontology and archaeology
☐	☒	Animals and other organisms
☒	☐	Clinical data
☒	☐	Dual use research of concern

Methods

n/a	Involved in the study	
☒	☐	ChIP-seq
☒	☐	Flow cytometry
☒	☐	MRI-based neuroimaging

Animals and other research organisms

Policy information about studies involving animals; ARRIVE guidelines recommended for reporting animal research, and Sex and Gender in Research

Laboratory animals	Five male C57BL6J mice were used in the study. The mice were bred in the Australian Phenomics Facility which were originally obtained from Jackson Laboratories. Mice were kept in cages attached to Tecniplast Smart Flow. The system keeps the cage temperature at 22 degree C but the humidity is not controlled but was typically around 40-50%. Mice were transfected at 4-5 weeks with recordings starting 4-5 weeks later, and lasted for 2 to 3 weeks. Mice were culled at a maximum of 12 weeks of age.
Wild animals	The study did not involve wild animals.
Reporting on sex	All mice were male.
Field-collected samples	The study did not involve samples collected from the field.
Ethics oversight	All methods were performed in accordance with the protocol approved by the Animal Experimentation and Ethics Committee of the Australian National University (AEEC 2012/64; 2015/74).

Note that full information on the approval of the study protocol must also be provided in the manuscript.
